# Two innovative solutions for fragmented care to multi-problem patients in deprived neighbourhoods: 2 case studies

**Published:** 2012-09-04

**Authors:** Jennifer R van den Broeke, Thomas Plochg, Karien Stronks

**Affiliations:** Academic Medical Center University of Amsterdam, The Netherlands; Academic Medical Center University of Amsterdam, The Netherlands; Academic Medical Center University of Amsterdam, The Netherlands

**Keywords:** generalist approach, population health thinking, systems thinking, deprived neighbourhoods, multi-problem patients

## Abstract

**Theory:**

In Western countries there is a growing awareness that the way in which health professionals work needs to be more responsive to patients’ and population health needs and problems [1] integrated care is seen as the way forward [2–4]. In the Netherlands low patient satisfaction and participation of multi-problem patients and high health care costs in two deprived neighbourhoods, initiated partnerships between one health insurer and two local authorities. Both partnerships led to cooperation between care, preventive services and social services (Amsterdam Noord and Utrecht Overvecht).

Little empirical research has been done regarding the ways in which collaboration of health and social services develops. There are some studies within which authors reflect on aspirations analogue to what the partnerships aspire to. From these insights we form a theoretical framework including ideas on generalist approach [1, 5–7] population health orientation [8–10], and on supporting self-management [11–13].

**Purpose:**

In two case studies we built a conceptual model in order to conceptualise this cooperation process in both neighbourhoods and explored the different structures both partnerships led to.

**Methods:**

In a collaborative research we constructed conceptual models and explored the realized structures. Data were drawn from literature review and exploration of the developmental process in both partnerships using qualitative methods (i.e. semi-structured interviews, focus group-interviews, document review, and observations).

**Results and conclusions:**

With the conceptual models we clarify the different paths taken in the two neighbourhoods, which resulted in two different solutions to fragmented care. We describe both structures the partnerships led to.

Powerpoint presentation available at http://www.integratedcare.org at congresses – San Marino – programme.
